# Melt-Processed Bioactive EVOH Films Incorporated with Ferulic Acid

**DOI:** 10.3390/polym13010068

**Published:** 2020-12-26

**Authors:** Alejandro Aragón-Gutiérrez, Estela Rosa, Miriam Gallur, Daniel López, Pilar Hernández-Muñoz, Rafael Gavara

**Affiliations:** 1 Grupo de Tecnología de Envases y Embalajes, Instituto Tecnológico del Embalaje, Transporte y Logística, ITENE, Unidad Asociada al CSIC, calle de Albert Einstein 1, 46980 Paterna, Valencia, Spain; estela.rosa@itene.com (E.R.); miriam.gallur@itene.com (M.G.); 2 Instituto de Ciencia y Tecnología de Polímeros, ICTP-CSIC, calle Juan de la Cierva 3, 28006 Madrid, Spain; daniel.l.g@csic.es; 3 Instituto de Agroquímica y Tecnología de Alimentos, IATA-CSIC, calle del Catedrático Agustín Escardino Benlloch 7, 46980 Paterna, Valencia, Spain; rgavara@iata.csic.es

**Keywords:** ferulic acid, EVOH copolymer, food packaging, film properties, antioxidant and antimicrobial packaging

## Abstract

In this work, antimicrobial and antioxidant films based on ethylene vinyl alcohol (EVOH) copolymer containing low amounts of ferulic acid (FA) were successfully developed by melt extrusion. Optically transparent films were obtained, and the presence of FA provided some UV blocking effect. The characterization of the thermal and barrier properties of the developed films showed that the addition of FA improved the thermal stability, decreased the glass transition temperature (T_g_) and increased the water vapor and oxygen transmission rates when ferulic acid was loaded above 0.5 wt.%, associated with its plasticizing effect. Mechanical characterization confirmed the plasticizing effect by an increase in the elongation at break values while no significant differences were observed in Young’s modulus and tensile strength. Significant antioxidant activity of all active films exposed to two food simulants, 10% ethanol and 95% ethanol, was also confirmed using the 2,2-diphenyl-1-pricylhydrazyl (DPPH) free radical scavenging method, indicating that FA conserved its well-known antioxidant properties after melt-processing. Finally, EVOH-FA samples presented antibacterial activity in vitro against *Escherichia coli* and *Staphylococcus aureus*, thus showing the potential of ferulic acid as bioactive compound to be used in extrusion processing for active packaging applications.

## 1. Introduction

Active food packaging technology based on the incorporation of active agents such as antimicrobials and antioxidants in the packaging material for their later migration into the food is an encouraging and rapidly emerging technology. It can provide the packed food with high quality, safety, and long shelf life, usually by inhibiting the lipid oxidation and reducing or retarding the growth of microorganisms [1]. Specifically, lipid oxidation can lead to the formation of off-odors and off-flavors, texture and color changes, which influences consumer choice and acceptance [2,3,4]. Nevertheless, not only are organoleptic changes important during the development of lipid oxidation, but also the formation of toxic compounds such as aldehydes and the loss of nutritional values [5]. In this context, current tendencies in active packaging include the addition of active natural agents (such as essential oils or polyphenol-rich extracts) into the polymeric packaging materials, restricting the utilization of synthetic additives and extending the shelf life through a controlled release of these active substances into the food [6,7,8,9]. The use of polymer-based packaging for food products shows many benefits compared to other materials used in packaging applications, such as paper, aluminum or glass, since plastic materials are more flexible in terms of reducing weight and energy requirements for their production [10]. Additionally, polymers are very attractive in active packaging applications since the well-established mass transport properties (permeation, sorption and migration) can constitute the mechanism of action of the active packaging system [1].

From the wide list of thermoplastic polymer materials currently used in food packaging applications, ethylene vinyl alcohol (EVOH) copolymer can be certainly considered as one of the most adequate for developing active systems because of its physicochemical properties. EVOH is a semicrystalline random copolymer with great chemical resistance, high transparency and outstanding gas barrier properties to hydrocarbons, specifically when a low content of ethylene (below 38% mol ethylene) is considered [11]. EVOH-based structures have been increasingly used in the food packaging applications, this sector being characterized by stringent standards in terms of gas and chemical resistance, aroma, water and hydrocarbon permeation. The mechanical and barrier properties of EVOH under dry conditions are attributed to the high inter- and intra-molecular cohesive energy and semicrystalline morphology [12]. The main drawback of EVOH copolymers is their moisture content sensitivity, which determines a substantial shrinkage of their features (thermal, mechanical and barrier properties) at high relative humidity environments [13]. However, this high affinity between EVOH and water, which results in severe plasticization of the copolymer when it is exposed to foods with high water activity, can be used as a triggering mechanism to allow the migration of active compounds previously incorporated in the polymer matrix to the food media [1].

Solution casting is probably the most common technique used to develop polymer-based active packaging materials; however, this procedure presents difficulties in order to be scaled up to an industrial level. Design of active plastics using conventional technologies such as melt extrusion technology has been also widely studied in the last years [14,15]. The major problem of this processing technique is the high temperature reached during processing, usually causing the decomposition or deactivation of these active compounds or even losses through volatilization, with the consequent decrease in the activity of extruded films. In this work, trans-ferulic acid was selected as bioactive compound to develop active materials with potential antioxidant and antimicrobial properties, taking advantage of its low volatility and high thermal stability [16]. Ferulic acid is a component of lignocelluloses and serves to cross-link lignin and polysaccharides, giving rigidity to cell walls. It is found in the seeds of plants such as rice, wheat, and oats [17]. While evidence of ferulic acid use as natural compound with potential antioxidant activity in food systems is already available [18,19], to the best of our knowledge, no evidence can be found describing the potential antimicrobial activity of ferulic acid in this specific sector. Furthermore, López-de-Dicastillo et al. incorporated ferulic acid at 5 wt.% into an EVOH matrix, and it was performed by a solvent-casting technique. Accordingly, this work aims to develop bioactive EVOH-based films that inhibit microbial growth and provide protection against oxidation in food products by incorporating low amounts (up to 1 wt.%) of trans-ferulic acid by melt extrusion processing. Considering that the high temperatures reached during extrusion processing may cause the deactivation of the bioactive ferulic acid compound, the antioxidant and antimicrobial properties of EVOH-based films were evaluated to corroborate the potential of ferulic acid as bioactive compound to be used in extrusion processing. Additionally, the effects of ferulic acid on the physicochemical, thermal, mechanical and barrier properties of EVOH-based materials were also investigated.

## 2. Materials and Methods

### 2.1. Materials

An ethylene vinyl alcohol copolymer (EVOH) with a 44% ethylene molar content was kindly provided by The Nippon Synthetic Chemical Company (Osaka, Japan). Trans-ferulic acid and 2,2-diphenyl-1-pricylhydrazyl 95% free radical were purchased from Sigma (Madrid, Spain). Ethanol and methanol were purchased from VWR International Eurolab (Llinars del Vallès, Barcelona). Water was obtained from an ultrapure water system Barnstead GenPure Pro (Thermo Fischer Scientific, Massachusetts, MA, USA).

For the microbiological assays: Gram-negative bacteria, *Escherichia coli* (*E. coli*) CECT 516 (ATCC 8739) and Gram-positive bacteria, *Staphylococcus aureus* (*S. aureus*) CECT 240 (ATCC 6538P) were obtained from the Spanish Type Culture Collection (CECT, Valencia, Spain) and selected for use in the antimicrobial tests because of their relevance in food packaging applications.

### 2.2. Thermal Stability of Trans-Ferulic Acid

Thermal stability of trans-ferulic acid was studied by thermogravimetric analysis (TGA) of FA powder by using a TGA Q-500 equipment (TA Instruments, New Castle, DE, USA) under nitrogen flow at a constant heating rate of 10 °C min^−1^. Differential scanning calorimetry (DSC) was performed under nitrogen atmosphere with a flow of 50 mL min^−1^ at a heating rate of 10 °C min^−1^ by using a DSC Q-2000 calorimeter (TA Instruments, New Castle, DE, USA). The software TA Instruments Universal Analysis 2000 was used to analyze and calculate the different thermal parameters from TGA, DTG and DSC curves.

### 2.3. Film Formation

EVOH–ferulic acid blend formulations were prepared by melt extrusion by means of an XPlore MC15 micro compounder (Xplore Instruments BV, Sittard, The Netherlands, 15 cm^3^ capacity), the operating temperatures being 180–185 and 190 °C. The processing was carried out under nitrogen atmosphere, the total residence time was 3 min, and the rotating screw speed was set at 100 rpm. Weight ratios of trans-ferulic acid with respect to the EVOH copolymer were 0.25, 0.5, 0.75 and 1 wt.%. The blends were subsequently injection-molded using a micro injector IM12 (Xplore Instruments BV, 12 cm^3^ capacity) to obtain different types of specimens: halter-type specimens for mechanical testing, and circular-shaped specimens for the antimicrobial assays. Films were also obtained by means of a homemade roll for the rest of the characterization purposes.

### 2.4. Quantification of Ferulic Acid in EVOH-Based Films after Processing

The amount of ferulic acid present in the EVOH-based films after processing was determined by solid–liquid extraction followed by liquid chromatography mass spectrometry (LC/MS) analysis. In brief, 1 cm^2^ of each film was extracted with 10 mL of methanol at 40 °C for 24 h. Three replicates were carried out for each formulation. An Ultivo triple quadrupole LC/MS (LC/TQ) instrument from Agilent Technologies (Agilent Technologies, Inc., Santa Clara, CA, USA), equipped with a 50 × 2.1 mm, 1.8 µm column was used. The mobile phase consisted of methanol/water 95: 5% *v/v* containing 0.1% ammonium hydroxide at 0.4 mL min^−1^ flow rate, the temperature was 23 °C and the injection volume was 15 μL. Calibration of ferulic acid was performed by injecting known amounts of the compound into the LC/MS instrument.

### 2.5. Structural and Morphological Properties of EVOH Blended with FA

#### 2.5.1. ATR-FTIR Analysis

Fourier transmission infrared spectra (FTIR) were obtained for all the samples using a TENSOR 27 Spectrophotometer (Bruker, Massachusetts, USA) in attenuated total reflectance (ATR) mode between 4000–650 cm^−1^ at room temperature, using 32 scans at a resolution of 4 cm^−1^. A background spectrum was acquired before each test to compensate by spectral subtraction the presence of CO_2_ or humidity effect.

#### 2.5.2. Morphological Analysis

A Hitachi S-4800 scanning electron microscope (Hitachi Ltd, Tokyo, Japan) with an accelerating voltage of 10 kV and a working distance of 15 mm was used to record SEM micrographs of the cross sections submitted to cryofracture of EVOH and EVOH–ferulic acid-based formulations. Samples were previously coated with a palladium/gold layer in vacuum conditions prior to their analysis to increase their electrical conductivity.

### 2.6. Thermal Characterization

#### 2.6.1. Differential Scanning Calorimetry (DSC)

DSC was used to determine the glass transition temperature (T_g_), melting temperature (T_m_), crystallization temperature (T_c_) as well as the enthalpies of the processes associated with melting and crystallization. The tests were carried out using a DSC Q-2000 calorimeter (TA Instruments, New Castle, DE, USA). The cooling and heating rates of the different cycles were 10 °C min^−1^ in nitrogen atmosphere, and the typical mass of the samples was 3.5 mg. The cycle program consisted of a first heating from ambient temperature to 225 °C, followed by a cooling to –50 °C and a second heating to 225 °C. The values of temperatures and enthalpies were obtained from the cooling cycle and the first and second heating cycles.

The crystallinity degree was calculated according to Equation (1):

(1) $$\chi \left(\%\right)=\frac{1}{\left(1-m_{f}\right)}\left[\frac{\Delta H}{\Delta H_{0}}\right]\times 100$$

where ΔH is the enthalpy for melting or crystallization; $\Delta H_{0}$ is melting for a 100% crystalline EVOH sample and $\left(1-m_{f}\right)$ is the weight fraction of EVOH in the sample. The melting temperature at 100% of EVOH was calculated according to Equation (2):

(2) $$\Delta H_{0}=\alpha \Delta H_{0}^{PVA}+\beta \Delta H_{0}^{PE}$$

where $\alpha \Delta H_{0}^{PVA}$ is enthalpy of melting for a 100% crystalline of polyvinyl alcohol (PVA) taken as 161.1 J g^−1^, while $\Delta H_{0}^{PE}$ is enthalpy of melting for a 100% crystalline of polyethylene (PE) taken as 290.0 J g^−1^ [20]. *α* and *β* are the molar fractions of vinyl alcohol (*α* = 0.56) and ethylene (*β* = 0.44) in EVOH. $\Delta H_{0}$ melting enthalpy for 100% crystalline EVOH is taken as 217.8 J g^−1^.

#### 2.6.2. Thermogravimetric Analysis (TGA)

TGA was carried out by dynamic measurements with TGA Q-500 thermogravimetric equipment at a constant heating rate of 10 °C min^−1^ under nitrogen atmosphere to avoid any thermo-oxidative degradation. The maximum degradation temperatures (T_max_) of the different degradation stages were calculated from the first derivative (DTG) of the TGA curves. The onset degradation temperature was calculated at a 5% weight loss (T_5%_) from the TGA curve.

### 2.7. Functional Properties of the Films

#### 2.7.1. Thickness and Optical Properties

The colorimetric changes provoked by the addition of different amounts of ferulic acid were evaluated based on the ASTM D 2244 [21], by measuring color coordinates in the CIELab color space: L* (lightness), a* (red-green) and b* (yellow-blue) were analyzed using a KONICA CM-2500d (Konica Minolta Sensing Americas, Inc., NJ, USA). The polar coordinates were obtained, and the chroma C* and the hue angle h° were calculated as follows:

(3) $$C^{*}=\sqrt{{\left(a^{*}\right)}^{2}+{\left(b^{*}\right)}^{2}};\;h^{{}^{\circ}}=\arctan\left(\frac{a^{*}}{b^{*}}\right).$$

The instrument was previously calibrated using a white and black standard tile. Total color differences caused by blending EVOH with ferulic acid with respect to the control EVOH material were evaluated using Equation (2). At least five readings were taken for each material and the average values were reported. The following assessment was used to evaluate the color change of the films based on the ∆E values: below 1 indicates an unnoticeable difference in color; 1–2 a slight difference that can only be noticed by an experienced observer; 2–3 a noticeable difference by an unexperienced observer; 3.5–5 a clear noticeable difference; and above 5, different colors are noticeable The yellowness index (YI) was also measured according to ASTM E313 [22].

(4) $$\Delta E=\sqrt{\Delta L^{2}+\Delta a^{*2}+\Delta b^{*2}}.$$

Light barrier properties of EVOH films after incorporating FA were tested by measuring the UV–visible light absorption spectra. For that, each film was cut into a rectangular piece (1 cm × 4 cm) and directly mounted between magnetic holders of a UV–Vis spectrophotometer Jasco V-630 (Jasco Deutschland GmbH, Pfungstadt, Germany) and the light absorbance of the films was read in the wavelength range of 200–800 nm. Film transparency was inversely given as apparent opacity by calculating the area under the absorption curve (Au × nm) in the visible wavelength (400–800 nm). The capacity of the films as filters of UV light was also evaluated by calculating the area under the absorption curve comprised between 200 and 399 nm.

#### 2.7.2. Water Contact Angle

Surface wettability was measured using a goniometer OCA15EC (DataPhysics Instruments, Filderstadt, Germany). For the measurement, each sample was placed on the horizontal movable stage of the contact angle analyzer. Four microliters of water drop was then added to the film surface using a micro-syringe. The captured images were studied using the SCA software and the contact angles for each analyzed sample were calculated. Five measurements were taken of each set of film samples, and the average values were taken.

#### 2.7.3. Barrier Properties

Water vapor transmission rate (WVTR) tests were conducted in accordance with ASTM F1249-01 [23], under tropical conditions, 38 °C and 90% RH using a Permatran-W Model 3/33 SG Plus Mocon (Lippke, Neuwied, Germany), while the oxygen gas transmission rates (OTRs) of the developed materials were determined at 0% RH and 23 °C, using an OX-TRAN Model 2/22 L OTR Analyzer Mocon, following the ASTM D3985-17 [24]. All tests were conducted in duplicate, and to perform the WVTR and OTR measurements, samples were conditioned in the permeation cells for 4 h, and the transmission rates were obtained once a constant value was reached.

#### 2.7.4. Mechanical Properties

The mechanical properties of the samples were obtained using a universal testing machine Testometric M350-20CT (Rochdale, UK), equipped with a 100 N load cell. The tests were carried out in accordance with UNE-EN ISO 527-2 [25] at a test speed of 20 mm min^−1^ and a distance between jaws of 50 mm. The dimensions of the halter-type specimens were 75 mm long, 4 mm wide and 2 mm thick. The results for Young’s modulus (E), tensile stress (TS) and deformation at break (ε_B_%) were calculated from the stress–strain curves as the average of at least five measurements.

### 2.8. Antioxidant Properties

#### 2.8.1. Antioxidant Activity of Films

The antioxidant activity of films was measured with a standard antioxidant assay using 2,2-diphenyl-1-pricylhydrazyl (DPPH), a stable radical that absorbs at 517 nm [26], following the method reported by Cejudo-Bastante et al. [27] but with slight modifications. In brief, a film sample (ca. 150 mg, size 10 × 10 mm) was immersed in 5 ml of a methanolic solution of stable DPPH radicals (50 mg L^−1^) and incubated in the dark. An aliquot (1 mL) was extracted at different times (1, 3, 18 and 24 h), measuring the absorbance of the DPPH solution at 517 nm. The antiradical activity was defined as the decrease in the absorbance of DPPH, and the percentage inhibition values were calculated according to Equation (5):

(5) $$I\;\left(\%\right)=\left(\frac{A_{c}-A_{s}}{A_{c}}\right)\times 100$$

where I (%) is the percentage of inhibition, *A_c_* is the absorbance of DPPH in methanolic solution and *A_s_* is the absorbance of DPPH after being in contact with each film sample at different times. Tests were carried out in duplicate and the results were expressed as I (%)/100 mg of film sample.

#### 2.8.2. Antioxidant Activity of Ferulic Acid Released into Food Simulants

Similarly, the antioxidant activity of the active EVOH-based films was evaluated in terms of the radical scavenging ability of the ferulic acid released into ethanol 10% (*v/v*) as an aqueous food simulant, and ethanol 95% (*v/v*) as a fatty food simulant, by using the DPPH method, as proposed by Ramos et al., with slight modifications [28]. Briefly, 3 cm^2^ of each film sample were immersed into 5 mL of the simulant (area-to-volume ratio around 6 dm^2^ L^−1^). After 24 h, 100 µL aliquots of each extract were mixed with 1.9 mL of a methanolic solution of DPPH (50 mg L^−1^) in a capped cuvette. The mixture was shaken vigorously and kept in the dark at room temperature for 30 min. All tests were performed in duplicate and the DPPH radical scavenging activity was expressed as % I according to Equation (5).

### 2.9. Antimicrobial Activity

Antibacterial assays were conducted according to the standard method JIS Z 2801 [29]. Figure S1 shows a schematic representation of the procedure followed to carry out the antibacterial assays. In brief, test pieces for the antibacterial assays were obtained by injection moulding and presented in circular shapes with 4 cm diameter and 1 mm thickness. The species of bacteria to be used for the test were *Staphylococcus aureus* and *Escherichia coli*. An overnight culture of these microorganisms was prepared by transferring a grown colony of *S. aureus* and *E. coli* into tubes containing 10 mL of Tryptic Soy Broth (TSB) culture medium and incubated at a temperature of 35 ± 1 °C for 17 h. From this culture, test inoculums were prepared, diluting appropriately with 1/500 nutrient broth so that the bacteria concentration became 2.5·10^5^ to 10·10^5^ colony forming unit (CFU)/mL. Then, each test piece was placed in a sterilized Petri dish making the test surface up; next, 0.3 mL of test inoculum was instilled onto each test piece. Petri dishes were covered with PP film (2.6 cm × 2.6 cm) and incubated at 36 °C for 24 h. After the incubation period, materials were transferred to a Stomacher pouch and were washed with 40 mL neutralizing solution (recipe: 34 g neutralizing broth base in 1 L distilled water with 5 mL Polysorbate 80, boiled and autoclaved). The serial dilutions of the rinse liquid were plated on plate count agar (PCA) using an automatic plater (easySpiral Pro, Interscience) to enumerate the viable cells in CFU (colony forming unit). Controls at 0 h and 24 h of inoculation were used and labelled as *U*_0_ and *U_t_*, respectively. Tests were performed in duplicate and the log reduction value, also called antibacterial activity value (R), was obtained according to Equation (6):

(6) $$R=Log\;U_{t}-Log\;A_{t}$$

where *U_t_* is the average of logarithm number of viable bacteria after inoculation on untreated test pieces after 24 h, and *A_t_* is the average of logarithm number of viable bacteria after inoculation on antibacterial test pieces after 24 h.

### 2.10. Statistical Analysis

Significance in the water contact angle data and mechanical properties was statistically analyzed by one-way analysis of variance (ANOVA) using OriginPro 8.6 software. Differences between average values were assessed on the basis of confidence intervals using the Tukey test at a confidence level of 95% (*p* < 0.05).

## 3. Results

### 3.1. Thermal Stability of Trans-Ferulic Acid

**Figure 1:**
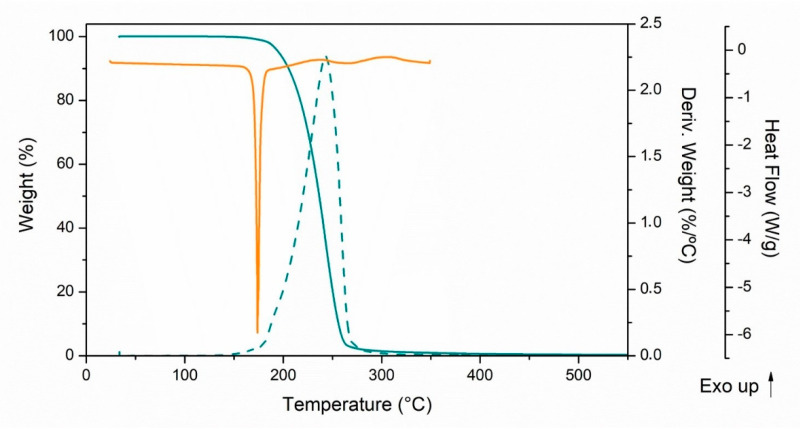
Thermogravimetric analysis (TGA) and DTG curves (cyan) and differential scanning calorimetry (DSC) thermogram (orange) of trans-ferulic acid.

Thermal properties of trans-ferulic acid were analyzed and Figure 1 shows the trans-ferulic acid curves of TGA/DTG analysis and the DSC thermogram during the first heating rate at 10 °C min^−1^. In the DSC curve it was possible to observe two endothermic peaks. The first and main one, located at 174 °C, was attributed to the melting process of ferulic acid, which is expected to occur between 173 and 176 °C. A second slight endothermic event appeared between 242 and 300 °C and was associated with FA degradation followed by the oxidation of carbonaceous matter, in good agreement with results obtained by TGA analysis.

The onset degradation temperature of trans-ferulic acid obtained from the TGA curve was 205 °C. This value is 15 °C above the processing temperature, positioning ferulic acid as a promising additive to be used in melt extrusion technologies. The DTG curve of trans-ferulic acid showed only one degradation step located between 200 and 300 °C with a mass loss of 98.9%. The peak, characterized by a high intensity centered at 243 °C, was attributed to the FA degradation products such as 4-vinylguaiacol, 4-methylguaiacol or guaiacol [30].

### 3.2. Quantification of Ferulic Acid in EVOH-Based Films

Five different formulations were obtained by melt extrusion processing and named as shown in Table 1. The quantification of the final amount of ferulic acid remaining in the active films after processing is essential since the high temperatures reached during the extrusion process may result in significant losses or degradation of the bioactive compound incorporated to the polymer matrix. The final concentration of ferulic acid (wt.%) was determined by LC-MS analysis for all binary formulations and the results are indicated in Table 1.

It was observed that the loss of ferulic acid during extrusion process was around 30%, regardless of the initial amount present in the formulations. Temperature, residence time of the mixture in the extruder, additive concentration, and the low volatility of ferulic acid caused by its high melting temperature (176 °C) were the main factors influencing its permanence in the final formulations. Based on previous works, optimization of the processing conditions (i.e., temperature profile, residence time, and screw speed rotation) is essential to achieve a good dispersion into the polymer matrix while avoiding unnecessary losses of bioactive compounds [31].

### 3.3. Structural and Morphological Properties of the Films

#### 3.3.1. FTIR Characterization

**Figure 2:**
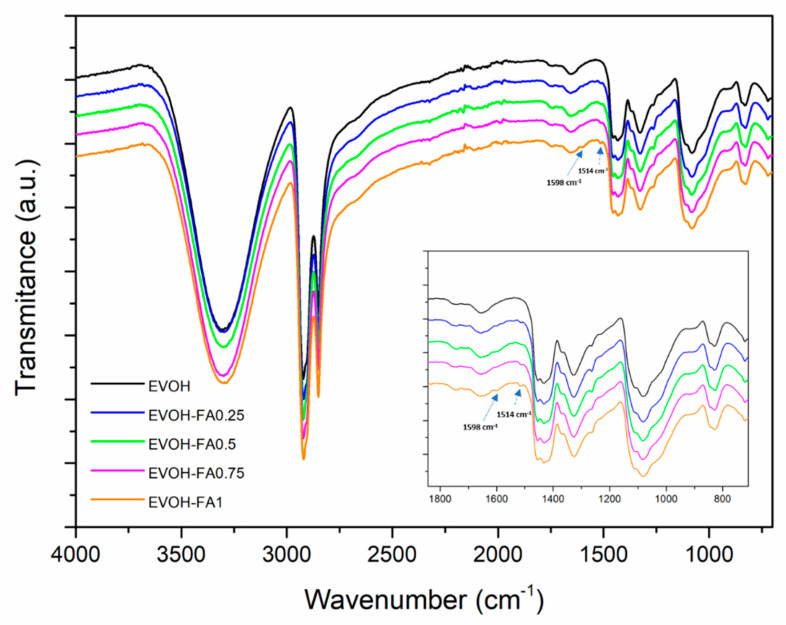
Fourier transmission **infrared** (FTIR) spectra of neat EVOH and EVOH samples incorporated with ferulic acid.

FTIR spectra of EVOH and EVOH–ferulic acid samples are shown in Figure 2, where the characteristic bands of the EVOH copolymer can be identified. Spectra have been shifted for better observation and the insert includes the spectra of films in the most relevant range. The main characteristic peaks of the bioactive trans-ferulic acid compound are located at 1512, 1601 cm^−1^ (νC=C), 1691 cm^−1^ (νC=O), 1202 cm^−1^ (νCO), 2943 cm^−1^ (ν_sym_CH*_n_*), 2963 cm^−1^ (ν_asym_CH) and 3440 cm^−1^ (νOH) [32,33]. Neat EVOH showed the absorbance bands characteristic in EVOH copolymers: adsorption peaks of stretching vibration at 2851 cm^−1^ (C–H), 2925 cm^−1^ (C–H) and 3340 cm^−1^ (O–H), and bending vibration at 1327 cm^−1^ (C–H) and 1450 cm^−1^ (C–H) [34,35]. As can be seen, no important differences were observed in the EVOH spectra caused by the addition of different amounts of ferulic acid. Only slight peaks appearing at 1514 and 1598 cm^−1^ could be detected and were associated with the νC=C of trans-ferulic acid. No other characteristic bands of ferulic acid were detectable in the spectra due to the low percentages of active compound in the formulations.

#### 3.3.2. SEM Studies

**Figure 3:**
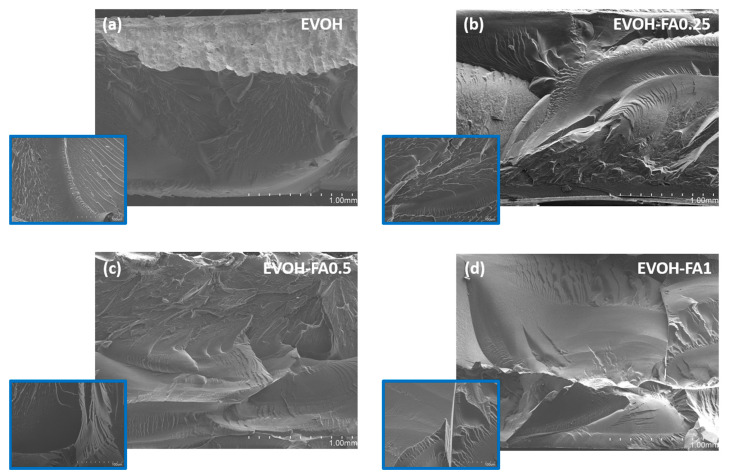
Cryofractured SEM micrographs of (**a**) EVOH, (**b**) EVOH-FA0.25 (**c**) EVOH-0.5FA and, (**d**) EVOH-FA1 at magnifications of 50× and 500× (inserts).

Morphological aspects of the cross cryofracture sections of film samples were evaluated by SEM and the images are shown in Figure 3. As expected, EVOH presented a smooth and uniform surface (Figure 3a) typically found in thermoplastic and well-processed semicrystalline polymeric materials. The addition of ferulic acid did not affect the aspect of cross sections in terms of homogeneity and uniformity.

This phenomenon could be related to the melting temperature of ferulic acid powder, which was located at 174 °C, close to the melting point of EVOH (165 °C) and in the same range of the processing temperature profile (180–185–190 °C). However, the presence of ferulic acid resulted in a surface with more roughness, presenting plastic deformation and showing more ductile fracture patterns (Figure 3b–d).

### 3.4. Thermal Analysis

#### 3.4.1. Differential Scanning Calorimetry

DSC thermal properties of EVOH–ferulic acid formulations obtained from the cooling and from the second heating rate are summarized in Table 2. Differential scanning calorimetry was employed to study the glass transition (T_g_), melting (T_m_) and crystallization (T_c_) of EVOH-based formulations. All samples showed a single glass transition temperature. Hydroxyl groups in the EVOH copolymer are strongly interconnected by hydrogen bonding, which contributes to a high value of T_g_. The introduction of other functional groups breaks down these hydrogen bonds and thus, the resulting T_g_ is affected by the modification degree and the character of the substituent group.

The addition of ferulic acid resulted in an evident decrease of T_g_ values with respect to the neat matrix. This reduction due to the addition of the natural antioxidant could be related with a higher mobility of the polymer macromolecules due to an increase in the free volume of the matrix, probably caused by a plasticizing effect of ferulic acid. T_c_ values during the cooling scan showed a slight decrease with the incorporation of ferulic acid, suggesting that this agent could favor the crystallization of EVOH at lower temperatures. Melting temperature values did not show significant differences when ferulic acid was incorporated.

Previous works related that the presence of different antioxidants probably produces two antagonistic effects: a nucleating effect on the polymer, which induces the growth of a large number of crystals, and a decrease in crystal size because of imperfections [18,36]. The type of processing usually results in changes in the thermal properties of EVOH copolymer materials. Interestingly, the addition of low amounts of ferulic acid did not produce changes in the degree of crystallinity of the EVOH copolymer. In this line, melt-processing promotes higher melting temperatures and melting enthalpy as compared to the solvent-casting method, since a more efficient crystallinity structure is achieved [13].

#### 3.4.2. Thermogravimetric Analysis

**Figure 4:**
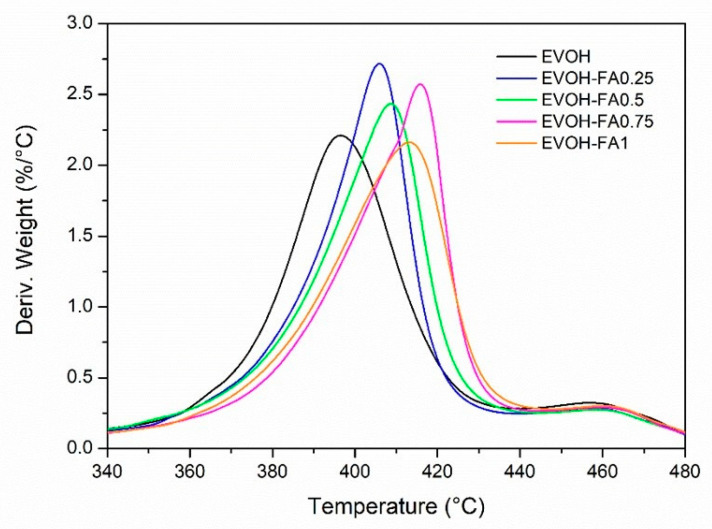
DTG curves of EVOH and EVOH-FA samples.

Table 3 summarizes the thermal parameters obtained from thermogravimetric analysis of the films. The temperature at maximum weight loss was obtained from the first derivative of the TGA curve (see Figure 4) which gives weight loss as a function of the temperature.

The addition of ferulic acid shifted for all formulations the onset degradation temperature to higher values, confirming the good ability of this natural antioxidant to stabilize EVOH. Trans-ferulic acid is a polyphenol whose chemical structure is characterized by several phenol rings and hydroxyl groups, which are able to act as natural stabilizers against the polymer degradation. Regarding the maximum degradation temperatures, all formulations presented two decomposition processes. The first degradation process in EVOH was centred at 397 °C, attributed to the major component of vinyl alcohol in the EVOH copolymer, while the second degradation process presented a maximum temperature peak centred at 456 °C, which was associated with the ethylene component in the polymer chain of EVOH [37].

No degradation process was found for ferulic acid, possibly due to its low contents added in the different formulations. The addition of ferulic acid shifted the maximum degradation temperatures of the first degradation stage to higher values in all blend formulations. EVOH-FA0.75 showed the highest T_onset_ and T_maxI_ values, leading to the highest thermal stable formulation. No significant differences were observed in the maximum peak temperature of the second degradation stage.

### 3.5. Functional Properties of Films

#### 3.5.1. Thickness and Optical Properties

Given the relevance of the visual aspect of the materials used in packaging applications, an analysis of the color properties was carried out to check whether the addition of ferulic acid into EVOH polymer matrix produced a noticeable optical effect. Figure S2 in the Supplementary Materials shows the visual appearance of the obtained materials. All film formulations were transparent to varying degrees, allowing one to see perfectly through the film.

Neat EVOH resulted in a colorless and transparent film while incorporation of ferulic acid slightly changed the chromaticity of EVOH to yellow tones. The visual appearance of the films was confirmed by means of colorimetric and light absorption measurements, and the results are displayed in Table 4. Neat EVOH showed great transparency along the visible region of the spectra (400–800 nm) while the addition of ferulic acid did not produce significant changes (see Figure S3 in the Supplementary Materials). This result was supported by calculating the area under the absorbance curve (Au × nm). EVOH film presented an area of 64 Au × nm, confirming its great transparency, while the incorporation of FA increased the area up to 82 Au × nm (EVOH-FA1), thus showing that FA did not have a substantial influence on the transparency properties of the films along the visible region. Regarding the UV spectra region (250–400 nm), ferulic acid produced a blocking effect. The ability of the developed films to act as filters of UV light was determined by calculating the area under the absorption curve comprised in the range of 200–399 nm. As reported in Table 4, the area under the curve along the UV range in the EVOH film was 181 Au × nm, while addition of FA provoked a raise above 1000 Au × nm in all formulations (up to 1113 Au × nm in EVOH-FA1). This effect was interesting since ferulic acid could not only prevent EVOH backbone deterioration, but also the reduction in UV transmission could minimize undesired photo-oxidation reactions in food [38].

Regarding the color values of EVOH and EVOH-FA films, neat EVOH was characterized by a high lightness value (L* = 89.6 ± 0.2). Interestingly, when ferulic acid was added to the EVOH copolymer, no significance differences in lightness values (L*) were observed. This result confirmed the high transparency of the developed films, as already observed by UV–Vis analysis, making it possible to see through the film without any problem, which is one of the most important requirements for consumers in food packaging applications. On the other hand, the presence of ferulic acid in EVOH-based formulations induced a color variation with respect to the neat extruded material. The EVOH copolymer had a* and b* values of 0.2 and 0.1, respectively. Positive values were registered in extruded EVOH combined with ferulic acid for b* parameter, indicating a deviation toward yellow tones, in good agreement with visual observations.

These changes were responsible for the total color difference values (ΔE) between 2 and 3.5, which means that changes are noticeable by an unexperienced observer. Additionally, yellowness index (YI) was also determined. The YI is used to describe the change in color of a sample from clear toward yellow. It was observed that the YI values increased with the ferulic acid content increase. However, the YI values obtained (ranging between 2.5 and 5) were similar to those that have been reported for other packaging plastic films, such as PET [39].

#### 3.5.2. Surface Wettability

The hydrophobic/hydrophilic nature of formulation surfaces was measured by means of static contact angle measurements. As mentioned before, EVOH is a copolymer consisting of polyethylene and vinyl alcohol. Neat LDPE has a contact angle (θ) around 99°, indicating a high surface hydrophobicity (contact angle > 90°) [40], while surface wettability of EVOH will depend on the molar fraction of each component in the copolymer. In the present study, the EVOH copolymer with a 44% mol of ethylene presented a water contact angle of 51° (Table 5). By the addition of FA, it was possible to observe an increase in the contact angle of all the developed formulations. The highest contact angle was obtained in EVOH-FA0.25, with a value of 71°. This result was related to the poor solubility of ferulic acid in water, which resulted in an increase of the surface hydrophobicity. However, the incorporation of higher amounts of ferulic acid caused a slight decrease of the contact angles with respect to the EVOH-FA0.25 formulation, suggesting that the introduction of the carboxyl groups of ferulic acid could be responsible for a higher interaction between water and the different polymer surfaces containing ferulic acid, decreasing in this way the hydrophobicity of EVOH–ferulic acid blends compared to EVOH-FA0.25.

#### 3.5.3. Mechanical and Barrier Properties

Tensile tests were conducted and results in terms of Young’s modulus (E), tensile strength (TS) and elongation at break (ε_B_) are summarized in Table 5

The addition of ferulic acid slightly increased the Young’s modulus, mainly maintaining the tensile strength, and at the same time a noticeable increase in the elongation at break was found for all formulations with respect to the EVOH sample, from 33% elongation of EVOH to 54% elongation for the EVOH-FA1 formulation, in good agreement with DSC analysis, where it could be observed that ferulic acid incorporation produced a plasticizing effect in the EVOH copolymer matrix, decreasing the glass transition temperature values.

Oxygen and water barrier properties are of great importance in packaging films intended to protect packaged products from the environment and maintain their quality for longer storage times. Since EVOH is one of the most commonly used gas barrier materials in food packages [41,42], it is important to evaluate how the addition of low amounts of ferulic acid has an influence on the barrier properties of the EVOH copolymer. Table 5 reports the OTR and WVTR values of all film samples. The addition of 0.25 wt.% and 0.5 wt.% of ferulic acid caused a decrease in OTR values of approximately 25% and 28%, respectively. However, once the concentrations were over 0.5 wt.% the OTR values increased again. This result was associated with the plasticizing effect of ferulic acid observed by DSC and elongation at break results, which increased the free volume in the EVOH matrix, leading to an increase in the mobility of polymer chains and, consequently, to an increment in the OTR values. Regarding the WVTR values, the addition of 0.25 wt.% of FA resulted in a slight decrease of WVTR with respect to the neat EVOH matrix, attributable to the poor solubility of ferulic acid in water. However, the noticeable plasticizing effect when ferulic acid concentrations were set over 0.25 wt.%, and the introduction of the hydroxyl groups present in ferulic acid could be responsible for the higher WVTR values observed in the formulations containing above 0.25 wt.% of ferulic acid, with an increase of up to 60% in the EVOH-FA1 sample.

### 3.6. Antioxidant Activity

**Figure 5:**
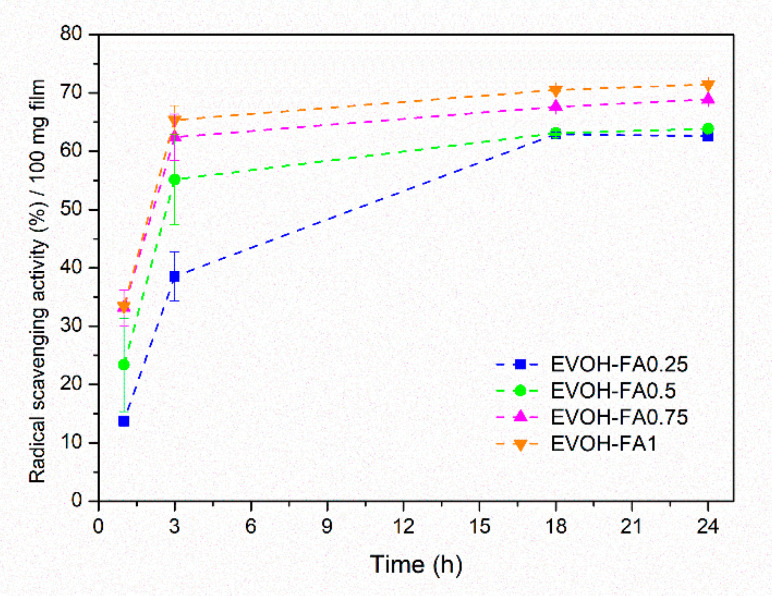
Radical scavenging percentages of EVOH films containing ferulic acid as a function of time. Each point represents the mean ± SD (*n* = 3).

The objective of this work was proving that the presence of antioxidant molecules in the polymer matrix conferred antioxidant activity to EVOH melt-extruded films. The mechanism of action of a food package design with the active films is based on the type of physicochemical behavior that occurs when a food product, its packaging and the environment are put in contact [43]. Antioxidant properties of EVOH films blended with FA was assured by the DPPH assay. In this test, a migration process occurs when film samples are immersed into methanolic solutions of DPPH. Figure 5 shows the antioxidant activity of EVOH films containing different quantities of ferulic acid expressed as the radical scavenging activity (RSA) (%) of the DPPH radical per 100 mg of film sample as a function of time. The results concluded that, as expected, the control of EVOH produced a null result (not included in the graph) while all the four active samples presented a clear radical scavenging activity in excess of the EVOH control.

The mechanism of action of ferulic acid to act as an antioxidant compound is based on the inhibition of the forming reactive nitrogen or oxygen species and on the neutralization of free radicals [44]. Even at the early stages (1–3 h), all formulations showed a significant increase of the radical scavenging percentage that may be attributed to the high reaction rate of the hydroxyl groups of ferulic acid and the DPPH free radicals. After 3 h of exposure, all active films except the EVOH-FA0.25 sample presented an RSA above 50%, while after 1 day all samples were able to scavenge between 60–70% of free radicals. This behavior can be explained by the interaction between methanol and EVOH-FA films producing a swelling of the polymer matrix, and on the other hand, by the high solubility of ferulic acid in this alcohol so that both of these effects can lead to a high release rate of ferulic acid into the DPPH solution over time.

**Figure 6:**
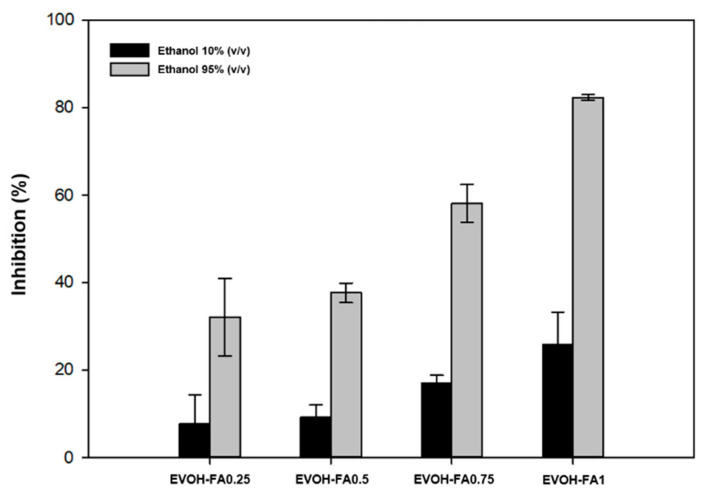
Antioxidant activity measured by the DPPH method in the two food simulants in contact with EVOH films containing ferulic acid.

Considering the significant antioxidant activity of the ferulic acid in the methanolic DPPH solution, the antioxidant properties of this compound released into two food simulants, ethanol 10% (*v/v*) and ethanol 95% (*v/v*), were also evaluated over one day using the DPPH method. Figure 6 shows the maximum radical scavenging activity after 24 h as a result of the release of ferulic acid into the two food simulants from the polymer matrix.

The antioxidant activity was found to be proportional to the ferulic acid concentration in the different simulants. In the 10% ethanol simulant, films with ferulic acid provided a low inhibition percentage value, with a maximum of around 25% inhibition in EVOH-FA1 film. The reason for this effect is the poor solubility of ferulic acid in this liquid media. In the 95% ethanol simulant, considerably greater antioxidant activity was reached by the films incorporated with FA compared to the activity in 10% ethanol. This result is in good accordance with the abovementioned higher solubility of this antioxidant in alcohols, and, additionally, by an increase in molecular mobility in the polymer produced by a plasticizing effect of ethanol, consequently improving the release of FA to the medium. These results are also in good agreement with previous reports dealing with bioactive polyphenolic compounds such as gallic acid or quercetin, presenting antioxidant capacity and strong protection of food when released from the polymer matrix [4,45,46,47]. Interestingly, the high processing temperatures did not cause the decomposition or deactivation of ferulic acid, maintaining its potential use in antioxidant packaging applications.

### 3.7. Antibacterial Activity

The antimicrobial activity of the developed films was tested against Gram-positive bacterium *S. aureus* and Gram-negative bacteria *E. coli.* Table 6 shows the results for the different EVOH–ferulic acid formulations. As shown, the incorporation of ferulic acid in the polymer matrix produced a growth reduction of both microorganisms in all formulations. A higher effectiveness was observed against *E. coli*, with reductions higher than 2 log from the 0.5 wt.% of ferulic acid, which is translated into an inhibition percentage higher than 99%. The addition of 1 wt.% of ferulic acid caused the highest decreased in the viable counts against *S. aureus*, with a maximum log reduction of 1.1.

In previous works, Borges, Ferreira et al. and Sorrentino, Succi et al. studied the antibacterial activity and mode of action of ferulic acid and gallic acid as phenolic compounds against pathogenic bacteria by determining different bacterial physiological indexes such as minimum inhibitory concentration (MIC), minimum bactericidal concentration (MBC), surface hydrophobicity and surface charge [48,49]. They concluded that the antimicrobial activity of ferulic acid could be associated with the affinity for the lipid bilayer in the cell membrane and the disruption of the membrane structure. In this sense, the phenolic acid–lipid interaction can help to explain the higher susceptibility of the Gram-negative bacteria. Indeed, the lipid content of the cell walls of the Gram-negative bacteria is substantially higher than that of the Gram-positive cell wall. Zeta potential measurements in the cited work also confirmed that the surface charge change was especially noticeable for the Gram-negative bacteria, demonstrating their higher susceptibility when compared to Gram-positive bacteria. In accordance with that study, in the present work was found that ferulic acid showed a higher effectiveness against *E. coli* than against *S. aureus*, but in all cases a decrease in the viable counts promoted by the addition of ferulic acid was achieved.

## 4. Conclusions

In the present work, novel active EVOH-based materials containing 0.25 wt.%, 0.5 wt.%, 0.75 wt.%, and 1 wt.% of bioactive trans-ferulic acid compound were prepared by melt extrusion processing in a twin-screw extruder and fully characterized. All film formulations resulted optically transparent, but ferulic acid produced a somewhat yellow tonality. However, absorbance studies revealed that ferulic acid provided a UV blocking effect to the EVOH films. EVOH-FA films presented improved thermal stability, and DSC analysis indicated a plasticizing effect of ferulic acid onto the EVOH matrix, resulting in a decrease in the T_g_. Mechanical properties supported the DSC results, since addition of ferulic acid resulted in a higher elongation at break while no differences were observed in Young’s modulus and tensile strength with respect to neat EVOH. An increase in WVTR and OTR values was observed when ferulic acid was incorporated above 0.5 wt.% and was attributed to the plasticizing effect of the bioactive compound in the EVOH copolymer. Despite this increase in the barrier property values, all active films showed positive results concerning antioxidant activity after melt extrusion processing, demonstrating high effectiveness in the radical scavenging inhibition using the DPPH method. That was one of the main objectives of the present work and, in addition to this, EVOH-FA samples exhibited antimicrobial activity against the Gram-negative *E. coli* and the Gram-positive *S. aureus* bacteria as compared to the pristine EVOH matrix. Higher effectiveness was found against *E. coli* with values of R > 2, which means that inhibition percentages higher than 99% were achieved. This result was attributed to the phenolic acid–lipid interaction, resulting in a higher susceptibility of the Gram-negative bacteria. The antimicrobial and antioxidant properties, together with the enhanced thermal stability, ductility, and the UV blocking effect in the developed formulations, as well as the possibility of being processed by melt compounding without occurring any degradation, are good indicators for the potential use of ferulic acid in active food packaging applications.

## Figures and Tables

**Table 1:** Film formulations and final amounts of ferulic acid quantified by liquid chromatography mass spectrometry (LC-MS) in the active films after melt extrusion processing (wt.%). Mean ± SD (*n* = 3).

Formulation	Code	Ferulic Acid (FA) (wt.%)
Ethylene vinyl alcohol (EVOH)	EVOH	Not detected
EVOH + FA 0.25 wt.%	EVOH-FA0.25	0.11 ± 0.01
EVOH + FA 0.5 wt.%	EVOH-FA0.5	0.37 ± 0.02
EVOH + FA 0.75 wt.%	EVOH-FA0.75	0.52 ± 0.05
EVOH + FA 1 wt.%	EVOH-FA1	0.71 ± 0.04

**Table 2:** Thermal parameters of EVOH and EVOH-FA samples obtained from DSC during cooling and second heating scans.

	Cooling			Second Heating			
Formulation	T_g_ (°C)	T_c_ (°C)	∆H_c_ (J/g)	T_g_ (°C)	T_m_ (°C)	∆H_m_ (J/g)	χ_c_ (%)
EVOH	50	145	67	54	165	70	31
EVOH-FA0.25	49	144	68	51	164	72	31
EVOH-FA0.5	47	143	63	50	163	64	29
EVOH-FA0.75	46	143	65	48	163	70	30
EVOH-FA1	45	143	65	48	163	69	30

**Table 3:** Onset degradation temperatures (T_5%_) and maximum degradation temperatures (T_max_) of EVOH and EVOH-FA films.

Formulation	T_5%_ (°C)	T_maxI_ (°C)	T_maxII_ (°C)
EVOH	348	397	457
EVOH-FA0.25	349	405	457
EVOH-FA0.5	349	408	458
EVOH-FA0.75	352	415	460
EVOH-FA1	351	413	460

**Table 4:** Thicknesses of films and color parameters obtained from CIELab space.

Formulation	Thickness (µm)	L* (D65)	C_ab_*	h°*_ab_*	ΔE	YI (E313)	UV (190–399 nm)	Visible (400–800 nm)
EVOH	83 ± 3	89.6 ± 0.2	0.2 ± 0.0	31.1 ± 0.8	-	-0.9 ± 0.1	181	64
EVOH-FA0.25	78 ± 5	89.5 ± 0.3	2.3 ± 0.2	96.3 ± 0.8	2.2 ± 0.1	2.7 ± 0.2	1036	61
EVOH-FA0.5	81 ± 4	90.3 ± 0.2	2.7 ± 0.2	98.7 ± 0.4	2.7 ± 0.2	3.3 ± 0.4	1066	63
EVOH-FA0.75	84 ± 7	89.8 ± 0.3	3.6 ± 0.2	96.4 ± 0.3	3.5 ± 0.2	4.7 ± 0.3	1062	68
EVOH-FA1	77 ± 3	90.2 ± 0.3	2.9 ± 0.3	95.1 ± 1.6	2.9 ± 0.2	3.7 ± 0.4	1113	82

L*: lightness; C_ab_*: chroma and h°*_ab_*: hue angle.

**Table 5:** Mechanical (mean ± SD; *n* = 5) and barrier properties and water contact angle measurements.

Formulation	E (GPa)	TS (MPa)	ε_B_ (%)	OTR (mL/m^2^ Day)	WVTR (g/m^2^ Day)	Θ (°)
EVOH	3.2 ± 0.0 ^a,b^	62 ± 1 ^a^	33 ± 3 ^a^	1.55 ± 0.26	0.20 ± 0.05	51 ± 4 ^a^
EVOH-FA0.25	3.2 ± 0.0 ^a^	61 ± 3 ^a^	38 ± 6 ^a,b^	1.16 ± 0.17	0.16 ± 0.02	71 ± 4 ^b^
EVOH-FA0.5	3.3 ± 0.1 ^b^	63 ± 2 ^a^	41 ± 2 ^a,b^	1.12 ± 0.11	0.23 ± 0.03	64 ± 2 ^b,c^
EVOH-FA0.75	3.3 ± 0.0 ^a,b^	60 ± 1 ^a^	43 ± 7 ^b^	1.91 ± 0.38	0.28 ± 0.04	62 ± 5 ^c^
EVOH-FA1	3.3 ± 0.0 ^b^	64 ± 2 ^a^	54 ± 4 ^c^	2.09 ± 0.56	0.32 ± 0.09	61 ± 6 ^c^

a–d: Different superscripts within the same column indicates significant differences between film formulations (*p* < 0.05).

**Table 6:** Antimicrobial effectiveness against *Escherichia coli* and *Staphylococcus aureus* of EVOH films containing 0.25, 0.5, 0.75 and 1 wt.% ferulic acid tested at 37 °C and expressed as logarithm of colony forming units (Log CFU) and log reduction value (R).

JIS Z 2801	*E. coli*		*S. aureus*	
Formulation	Log CFU/cm^2^ Mean	Antibacterial Activity (R)	Log CFU/cm^2^ Mean	Antibacterial Activity (R)
EVOH	6.66 ± 0.12	-	5.16 ± 0.00	-
EVOH-FA0.25	4.99 ± 0.69	1.6	5.22 ± 0.12	-
EVOH-FA0.5	4.01 ± 0.11	2.6	4.49 ± 0.24	0.7
EVOH-FA0.75	3.47 ± 0.11	3.2	4.34 ± 0.22	0.8
EVOH-FA1	2.97 ± 0.08	3.7	4.03 ± 0.22	1.1

## Supplementary Materials

The following are available online at https://www.mdpi.com/2073-4360/13/1/68/s1, Figure S1: Schematic representation of JIS Z 2801 standard method; Figure S2: Visual aspect of the samples; Figure S3: Light transmission of the films in the range of 250–800 nm.
