# Supplementary material for: Melt-Processed Bioactive EVOH Films Incorporated with Ferulic Acid
Source: Polymers (Basel). 2020 Dec 26;13(1):68. doi: 10.3390/polym13010068 (PMC7795252; doi:10.3390/polym13010068)
Supplement: Supplementary file 1 [file polymers-13-00068-s001.pdf]

SUPPORTING INFORMATION

Melt-processed Bioactive EVOH Films Incorporated with Ferulic Acid

Alejandro Aragón-Gutiérrez [1,*], Estela Rosa [1], Miriam Gallur [1], Daniel López [2], Pilar Hernández-Muñoz [3,*] and Rafael Gavara [3]

[1] Grupo de Tecnología de Envases y Embalajes, Instituto Tecnológico del Embalaje, Transporte y Logística, ITENE, Unidad Asociada al CSIC, calle de Albert Einstein 1, 46980 Paterna, Valencia, Spain; estela.rosa@itene.com (E.R.); miriam.gallur@itene.com (M.G.)

[2] Instituto de Ciencia y Tecnología de Polímeros, ICTP-CSIC, calle Juan de la Cierva 3, 28006 Madrid, Spain; daniel.l.g@csic.es

[3] Instituto de Agroquímica y Tecnología de Alimentos, IATA-CSIC, calle del Catedrático Agustín Escardino Benlloch 7, 46980 Paterna, Valencia, Spain; phernan@iata.csic.es; rgavara@iata.csic.es

* Correspondence: alejandro.aragon@itene.com (A.A.-G.); phernan@iata.csic.es (P.H.-M.); Tel.: +34-961 820 000 (A.A.-G.); +34-963 900 022 (P.H.-M.)

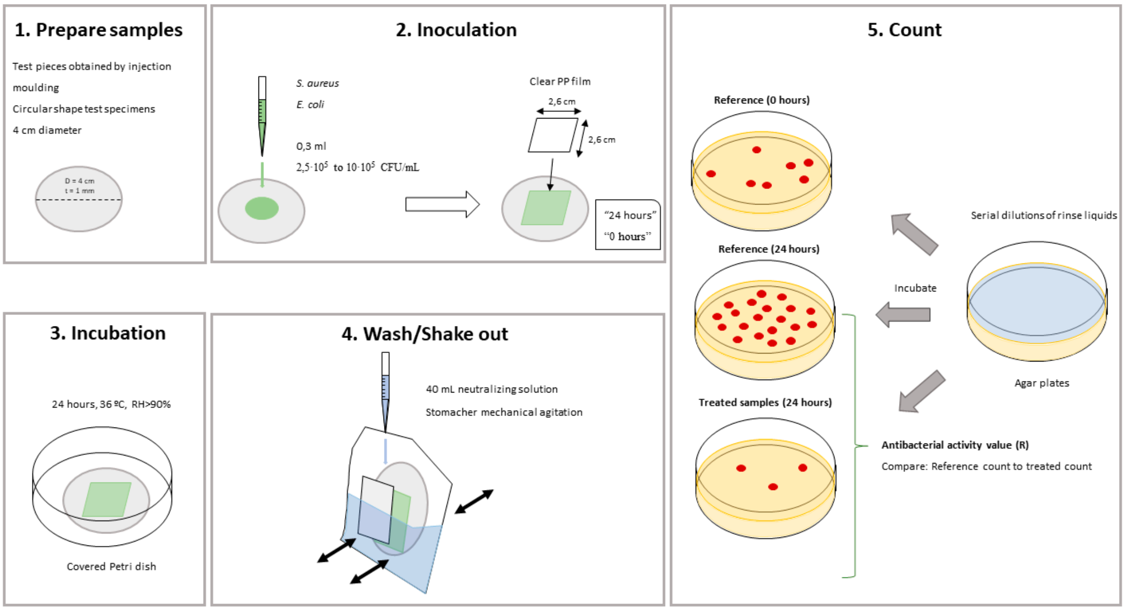

Figure S1. Schematic representation of JIS Z 2801 standard method.

****

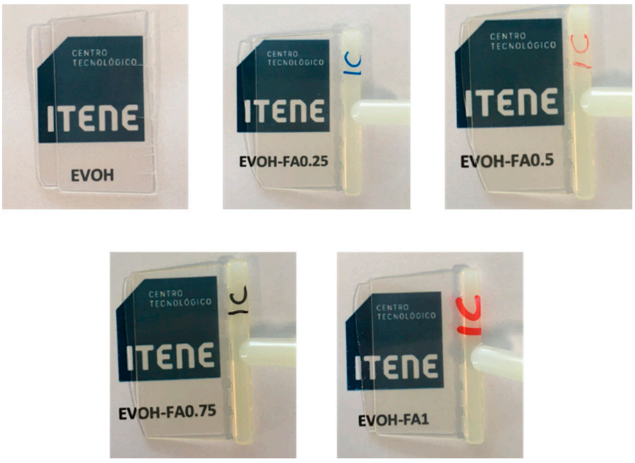

Figure S2. Visual aspect of the samples

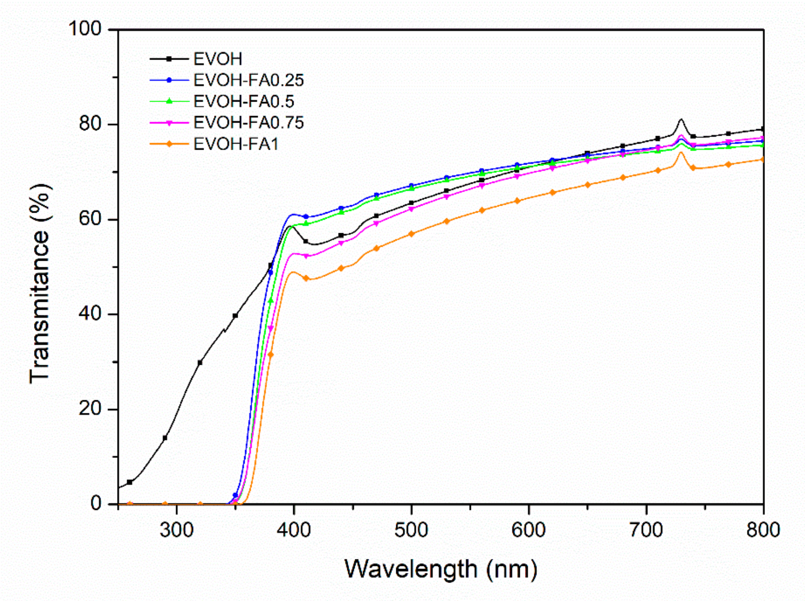

Figure S3. Light transmission of the films in the range of 250-800 nm

****
